# Performance of cotton expressing Cry1Ac, Cry1F and Vip3Aa19 insecticidal proteins against *Helicoverpa armigera, H. zea* and their hybrid progeny, and evidence of reduced susceptibility of a field population of H. zea to Cry1 and Vip3Aa in Brazil

**DOI:** 10.1371/journal.pone.0289003

**Published:** 2023-07-25

**Authors:** Luiz H. Marques, Tamylin K. Ishizuka, Renata R. Pereira, Ademar N. Istchuk, Jaedino Rossetto, Valeria F. Moscardini, Oscar A. N. B. e Silva, Antonio C. Santos, Timothy Nowatzki, Mark L. Dahmer, Amit Sethi, Nicholas P. Storer, Pablo C. Gontijo, Jacob C. Netto, Marlon A. G. Weschenfelder, Poliana G. de Almeida, Oderlei Bernardi

**Affiliations:** 1 Corteva Agriscience, Mogi Mirim, SP, Brazil; 2 Corteva Agriscience, Johnston, IA, United States of America; 3 Instituto Federal Goiano (IF Goiano), Campus Rio Verde, Rio Verde, GO, Brazil; 4 Instituto Mato-grossense do Algodão (IMAmt), Primavera do Leste, MT, Brazil; 5 Federal University of Santa Maria (UFSM), Santa Maria, RS, Brazil; Nigde Omer Halisdemir University, TURKEY

## Abstract

The genetically modified cotton DAS-21023-5 × DAS-24236-5 × SYN-IR102-7 expressing Cry1Ac, Cry1F and Vip3Aa19 from *Bacillus thuringiensis* Berliner (Bt) has been cultivated in Brazil since the 2020/2021 season. Here, we assessed the performance of DAS-21023-5 × DAS-24236-5 × SYN-IR102-7 cotton expressing Cry1Ac, Cry1F and Vip3Aa19 against *Helicoverpa armigera* (Hübner), *Helicoverpa zea* (Boddie), and their hybrid progeny. We also carried out evaluations with DAS-21023-5 × DAS-24236-5 cotton containing Cry1Ac and Cry1F. In leaf-disk bioassays, DAS-21023-5 × DAS-24236-5 × SYN-IR102-7 was effective in controlling neonates from laboratory colonies of *H*. *armigera*, *H*. *zea* and the hybrid progeny (71.9%–100% mortality). On floral bud bioassays using L2 larvae, *H*. *zea* presented complete mortality, whereas *H*. *armigera* and the hybrid progeny showed <55% mortality. On DAS-21023-5 × DAS-24236-5 cotton, the mortality of *H*. *armigera* on leaf-disk and floral buds ranged from 60% to 73%, whereas mortality of hybrids was <46%. This Bt cotton caused complete mortality of *H*. *zea* larvae from a laboratory colony in the early growth stages, but mortalities were <55% on advanced growth stages and on floral buds. In field studies conducted from 2014 to 2019, DAS-21023-5 × DAS-24236-5 × SYN-IR102-7 cotton was also effective at protecting plants against *H*. *armigera*. In contrast, a population of *H*. *zea* collected in western Bahia in 2021/2022 on Bt cotton expressing Cry1 and Vip3Aa proteins, showed 63% mortality after 30 d, with insects developing into fifth and sixth instars, on DAS-21023-5 × DAS-24236-5 × SYN-IR102-7 cotton. We conclude that *H*. *armigera*, *H*. *zea*, and their hybrid progeny can be managed with DAS-21023-5 × DAS-24236-5 × SYN-IR102-7 cotton; however we found the first evidence in Brazil of a significant reduction in the susceptibility to DAS-21023-5 × DAS-24236-5 × SYN-IR102-7 cotton of a population of *H*. *zea* collected from Bt cotton in Bahia in 2021/2022.

## Introduction

*Helicoverpa* spp. is a group of harmful agricultural pests in several regions of the world. In Brazil, the most important *Helicoverpa* species are the Old World bollworm, *Helicoverpa armigera* (Hübner)—first recorded in 2013 in soybean in Goiás and Bahia states—, and the corn earworm, *Helicoverpa zea* (Boddie) (Lepidoptera: Noctuidae) [1–3]. In the current Brazilian crop production systems, outbreaks of *Helicoverpa* species are favored by the overlapping and simultaneous cultivation of host plants, including soybean, maize, cotton, tomatoes, and dry bean crops [4]. Studies also reported the occurrence of interspecific hybridization between *H*. *armigera* and *H*. *zea* under laboratory conditions [5–7]. The successful hybridization in a restricted environment suggests that potential for multiple mating events in the field may provide opportunities to select well-adapted hybrid phenotypes [7]. Cases of resistance to Bt proteins in *H*. *zea* have been reported in the U.S. and China [8–11] and decreases in susceptibility to Cry proteins by *H*. *armigera* have been reported in U.S., India, and China [12, 13].

In Brazil, *H*. *armigera* has great importance in soybean and cotton [1, 4], while *H*. *zea* was most abundant in maize and cotton [14, 15]. The adoption of genetically-modified (GM) plants expressing insecticidal proteins derived from the soil bacterium *Bacillus thuringiensis* Berliner (Bt) is the main management tactic used against *Helicoverpa* species in cotton, soybean and maize fields in Brazil [16, 17]. The successful adoption of Bt crops has enabled more effective management of lepidopteran pests and reduced chemical insecticide applications in these crops worldwide [18–20].

In Brazil, Bt cotton was planted commercially for the first time in 2006. Transgenic cotton technologies are cultivated on approximately 1.4 million hectares (90% of the total area with Bt cotton) in recent cotton seasons [21]. The first generation of commercial Bt plants expressed single Bt proteins, but low compliance with resistance management strategies (e.g. refuge areas) in areas cultivated with Bt maize has favored the cross-crop resistance, affecting the performance of other Bt plants, including Bt cotton varieties [22]. The second and third generations of Bt cotton met the concept of ‘gene pyramiding’ with the expression of two or more Bt proteins with high toxicity and distinct modes of action to the same pest species [23, 24]. For example, the DAS-21023-5 × DAS-24236-5 × SYN-IR102-7 event (commercially named WideStrike^®^ 3) is part of the third generation of Bt cotton cultivated in Brazil and expresses Cry1Ac, Cry1F and Vip3Aa19 insecticidal proteins [25].

Previous studies found that WideStrike^®^ 3 was effective against *Spodoptera frugiperda* (J. E. Smith, 1797), *Spodoptera cosmioides* (Walker, 1858), *Chloridea virescens* (F., 1781), *Chrysodeixis includens* (Walker, 1858), and *Alabama argillacea* (Hübner, 1823) (Lepidoptera: Noctuidae) [25]. However, the performance of the WideStrike^®^ 3 was not reported against *Helicoverpa* species. On the present study, we evaluated the performance of Bt cotton DAS-21023-5 × DAS-24236-5 × SYN-IR102- expressing Cry1Ac, Cry1F and Vip3Aa19 and DAS-21023-5 × DAS-24236-5 that expresses Cry1Ac and Cry1F against *H*. *armigera*, *H*. *zea* and their hybrid progeny. We performed evaluations in laboratory and field trials across several cotton planting regions in Brazil. We also investigated the susceptibility to Bt cotton expressing Cry and Vip3Aa insecticidal proteins in a population of *H*. *zea* collected from Bt cotton expressing the Cry1Ab, Cry2Ae, and Vip3Aa Bt proteins in Bahia, Brazil.

## Material and methods

### A. Description of pest species and hybridization crosses

The two *Helicoverpa* species, *H*. *armigera* and *H*. *zea*, were collected from the field and maintained at Corteva Agriscience insectary (Toledo, Paraná, Brazil). Larvae of *H*. *zea* were collected from corn ears and *H*. *armigera* from soybean plants in Brasilia, DF during the 2015 crop season. Insects were maintained on an artificial insect diet proposed by Greene et al. [26], being that new field-collected larvae from non-Bt maize (*H*. *zea*) and non-Bt cotton or non-Bt soybean (*H*. *armigera*) were introduced to the colonies every year (~500 larvae/year). The new field larvae were collected in Paraná, São Paulo or Goiás states from 2016 to 2019. Hereafter, we refer to these populations as Hz-Lab and Ha-Lab. To design hybridization crosses, adults were confined in a PVC cage (30 cm in diameter × 30 cm height). Only the crossing between (500 ♀ Ha-Lab × 500 ♂ Hz-Lab) produced eggs that produced neonates. Hybrid larvae (F_1_ generation) were tested in confined laboratory conditions, to avoid any escapes to the environment.

In addition, during the 2021/2022 cotton season, a field population of *H*. *zea* was collected in Bt cotton expressing Cry1Ab, Cry2Ae and Vip3Aa (variety FM 985, FiberMax® - BASF S.A., São Paulo, SP, Brazil) in Luis Eduardo Magalhães, BA (latitude 12°07’29” S, longitude 46°08’28” W). After the collection, this population was transferred to an artificial diet [26] and maintained in a laboratory at Instituto Mato-Grossense do Algodão (IMAmt), Primavera do Leste, MT, Brazil. We refer to this population as Hz-field.

Before starting bioassays, all species were confirmed by PCR testing according to the methodology described by Perera et al. [27].

### B. Leaf-disk and square bioassays in laboratory trials

Leaf-disk and square bioassays were performed at Corteva Agriscience site located in Mogi-Mirim, SP, Brazil. The cotton technologies evaluated were: 1) A Bt cotton variety (TMG 91WS3 –full Maturity, Tropical Melhoramento & Genética S.A.–Cambé, Paraná, Brazil) containing events DAS-21023-5 × DAS-24236-5 × SYN-IR102-7 (WideStrike^®^ 3 Insect Protection, Corteva Agriscience, Indianapolis, IN) expressing Cry1Ac, Cry1F and Vip3Aa19 Bt proteins; 2) A Bt cotton variety (TMG 81WS–full Maturity) containing events DAS-21023-5 × DAS-24236-5 (WideStrike^®^ Insect Protection, Corteva Agriscience, Indianapolis, IN) expressing Cry1Ac and Cry1F Bt proteins, and 3) A non-Bt cotton variety (FMT 701, Fundação Mato Grosso, Rondonópolis, MT, Brazil). Weekly plantings were done under field conditions for two months to have supply of fresh leaves and squares at the same time. Before the bioassays, Bt and non-Bt plants were checked to confirm Bt protein expression using detection kits for Cry1Ac, Cry1F and Vip3A proteins (Envirologix, São Paulo, SP, Brazil).

Bioassays were performed with leaf disks of Bt and non-Bt cotton varieties previously described from leaves excised of plants in vegetative (growth stage 15 – 5th true leaf unfolded) and reproductive (growth stage 55 –squares distinctly enlarged) stages according to the phenological scale of Munger et al. [28]. Squares (bracts + buds) from a reproductive stage (growth stage 55) were also tested. Leaves and squares were removed from the upper third of the plants when they reached the respective phenological stage. Leaf disks 1.2 cm in diameter were cut using a metallic cutter. Leaf disks and squares were placed over a gelled mixture of water-agar 2.5% (1 ml/well) in 128-well bioassay trays (BIO-BA-128; CD International Inc., Pitman, NJ). The vegetative and reproductive structures tested were separated from the water-agar layer by a filter paper disk. Then, a single neonate (Ha-Lab, Hz-Lab and their F_1_ hybrid progeny) was placed on each leaf disk or a single L_2_ larvae was placed on each square. The trays were sealed with self-adhesive plastic sheets (BIO-CV-16; CD International Inc.) that allowed for gas exchange and then placed in a climatic chamber (temperature 25 ± 2°C, 60 ± 5% RH, and 14:10 h photoperiod). The experimental design was completely randomized with 8 replicates/growth stage/cotton technology (each replicate was represented by 16 larvae). Mortality, damage on leaf disks and squares, and larval weight were recorded at 5 d after infestation. The damage on each leaf disk was recorded using two methods. We measured the area (cm^2^) that was consumed by larva using a transparent grid ruler, and we also estimated the damage as percentage of the disk that was consumed. The damage on squares was classified as a surface feeding on the external side of the square, and as bored squares, when the larvae could feed inside the square. All live larvae in each replicate were pooled to obtain the weight of the replicate and recorded, since some larvae were too small to accurately weigh individually.

### C. Survivorship of *Helicoverpa* species on cotton throughout the larval cycle

Leaves of Bt and non-Bt cotton varieties previously described were excised from greenhouse-grown plants at the growth stage 15, according to the scale of Munger et al. [28]. In the laboratory, leaves were cut into pieces (6–8 cm^2^) and placed on a gelled mixture of agar-water at 2.5% in 50-mL plastic cups. Then, a single neonate from the Ha-Lab or Hz-Lab populations was placed in each cup, with leaves replaced every 24 h. Cups were placed in a climatic chamber at 25 ± 2°C, 60 ± 5% RH, and 14:10 h photoperiod. The bioassays with Ha-Lab and Hz-Lab populations were performed at Federal University of Santa Maria, RS, Brazil.

Similar bioassays were performed with the Hz-field population at Instituto Mato-Grossense do Algodão, Primavera do Leste, MT, Brazil. In bioassays, leaves were removed from cotton plants at the growth stage 15, cut into pieces and placed in 24-well plastic plates. Then, a single neonate (F_1_ generation) was placed in each well. When larvae reach 3^rd^ instar, they were individualized in Petri dishes (10 cm diameter × 1.5 cm height), with leaves replaced daily, being maintained in the same environmental conditions previously described. The cotton varieties tested in bioassays using the Hz-field population were: 1) TMG 50WS3 (early Maturity–Tropical Melhoramento & Genética S.A.–Cambé, Paraná, Brazil) containing events DAS-21023-5 × DAS-24236-5 × SYN-IR102-7 expressing Cry1Ac, Cry1F and Vip3Aa19 transgenic proteins; 2) TMG 81WS containing events DAS-21023-5 × DAS-24236-5 expressing Cry1Ac and Cry1F proteins; and 3) non-Bt cotton (IMA 2106 GL–mid Maturity, Instituto Mato-Grossense do Algodão, Cuiabá, MT, Brazil).

Before bioassays, plants were checked for the presence of the expected Bt proteins using detection kits (Envirologix, QuickStix, São Paulo, SP, Brazil) for Cry1Ac, Cry1F and Vip3A proteins. The experimental design was completely randomized with 42 and 26 replicates of 10 neonates for Bt cotton treatments, respectively, and 13 replicates of 10 neonates for the non-Bt cotton in bioassays with Ha-Lab and Hz-Lab, whereas 5 replicates of 20 neonates/cotton variety was used in bioassays with Hz-field. For both species, larval survival was evaluated every 5 d.

### D. Field efficacy of Bt cotton against artificial and natural infestations of *H*. *armigera*

Ten field experiments were conducted from 2014 to 2019 growing seasons across four different states in Brazil (Table 1). All trials were conducted followed strict adherence to Brazilian regulatory requirements in accredited and certified field research stations operated by Corteva Agriscience or SGS Company. Field trials were performed under regulated permits approved by the Comissão Técnica Nacional de Biossegurança (CTNBio). Treatments included were: 1) A Bt cotton variety containing events DAS-21023-5 × DAS-24236-5 × SYN-IR102-7 (WideStrike® 3 Insect Protection) expressing Cry1Ac, Cry1F and Vip3Aa19 Bt proteins; 2) A Bt cotton variety containing events DAS-21023-5 × DAS-24236-5 (WideStrike® Insect Protection) expressing Cry1Ac and Cry1F Bt proteins, and 3) A non-Bt isoline cotton variety with a similar genotypic background and belonging to the same maturity group as the Bt cotton varieties. The Bt cotton variety used was PHY 440 (Mid-full Maturity, PhytoGen^®^ Cotton Seeds) in all treatments. Each field trial consisted of four replications for each treatment arranged in a randomized complete block design. Plot size varied among locations from 5 to 8 meters in length and 5 or 7 rows wide. Row spacing in all locations varied from 50 to 76 cm.

**Table 1:** Field trial locations and geographic coordinates for each study performed from 2014 to 2019 in Brazil.

Location (city, state)	Geographic coordinates	Year	Infestation
Indianópolis, MG	18°57′29.70′′ S; 47°51′21.10′′ W	2014	*Helicoverpa armigera* artificial infestations
	18°57′24.69′′ S; 47°51′12.09′′ W	2016	
	18°57′08.60′′ S; 47°51′11.80′′ W	2016	
	24°47′14.20′′ S; 49°51′02.00′′ W	2018	
Montividiu, GO	17°22′40.4′′ S; 51°23′39.36′′ W	2014	
Palotina, PR	24°21’43.00" S; 53°45’23.70" W	2016	
Rio Verde, GO	17°45′02.20″ S; 51°02′18.30″ W	2016	
	17°45′27.40″ S; 51°02′03.70″ W	2017	
	17°45′18.20″ S; 51°02′08.10″ W	2018	
Mogi Mirim, SP	22°26’49.20" S; 47°04’09.90" W	2019	
Rio Verde, GO	17°45′02.20″ S; 51°02′18.30″ W	2016	*Helicoverpa* spp. natural infestation

#### Field efficacy of Bt cotton against artificial infestations of *H*. *armigera*

For this study, only *H*. *armigera* was infested in the field trials because previous laboratory trials on leaf disks with *H*. *zea* showed complete mortality. Larvae of *H*. *armigera* were obtained from laboratory-reared colony maintained by Corteva Agriscience (Mogi Mirim Research Center, Mogi Mirim, São Paulo State, Brazil) or SGS Company (Piracicaba, SP, Brazil). Artificial infestations were conducted at two different cotton reproductive growth stages at all locations to ensure uniform pest pressure across experimental plots (Table 1). The first artificial infestation was conducted at GS6: 65, at the beginning of flowering (“mid bloom”), and the second infestation at GS6: 65+, 10–12 d later [28]. For each plot, ten plants were randomly selected and each one was infested with 10 L1 larvae. Larvae were placed on the growing points of the selected plants, and then covered immediately with mesh cages (150 cm long × 50 cm wide × 150 cm high) to limit larval escape and to avoid mortality caused by natural enemies. Field evaluations consisted of recording the total number of cotton squares on 10 infested plants, the percentage of damaged squares, and the number of live larvae still present. These evaluations were performed 10 d after each infestation.

#### Field efficacy of Bt cotton against natural infestation of *Helicoverpa spp*

A natural infestation of *Helicoverpa* spp. occurred only in one trial at Rio Verde (GO) (Table 1). At this location, plot inspections and evaluations were performed weekly. The data presented in this paper represent the sampling date when the peak number of damaged squares and the number of live larvae were recorded for the non-Bt treatment.

### E. Data analysis

The number of insects tested and the number dead in leaf-disk and square bioassays, and survivorship of Ha-Lab, Hz-Lab and Hz-field throughout the larval cycle on Bt and non-Bt cotton leaves were used to estimate 95% confidence intervals (CIs) for the probability of mortality, according to a binomial distribution [29]. To perform these analyses, the function *binom*.*probit* from the package *binom* in R 3.1.0 [30] was used. Percent mortality rate on Bt and non-Bt cotton were declared significantly distinct if 95% CIs did not overlap [31, 32].

The variables of larvae developing on Bt and non-Bt cotton technologies (consumed leaf area, percent of leaf consumption, bud damage, and larval weight) and data from field trials (artificial infestation) on the efficacy of Bt cotton technologies against *H*. *armigera* were analyzed using a linear mixed model (PROC MIXED procedure) and statistical significance were obtained by using Tukey’s Honestly Significant Difference test with α = 0.05 in SAS [33]. Prior to analysis, data from each trial were evaluated to ascertain its validity by assessing the injury level in the non-Bt control. Trials selected for analysis only included those in which the non-Bt control averaged ≥10% square injury. Furthermore, prior to the combined analysis, each trial was analyzed individually and the mean square error (MSE) of the residual was used to evaluate the homogeneity of the variance error. Only trials that showed a ratio between the largest and smallest MSE ≤7 were included in the combined cross-trial analyses [34]. This procedure ensured that trials were homogeneous to avoid bias caused by differences among trials (sites and years). The data from the single, naturally-infested location of *Helicoverpa* spp. was subjected to *t*-test (PROC TTEST procedure) paired with α = 0.05 in SAS [33]. To improve the normal distribution, data of squares injured were log (x + 1) transformed, while data on number of larvae were transformed using $\sqrt{x+1}$. Non-transformed data are presented in figures.

## Results

### A. Leaf-disk and square bioassays

#### Mortality on leaf-disk and square

No significant differences were observed on mortality at 5 d for *H*. *armigera* neonates (Ha-Lab) on leaf-disks (67.2%–85.9%) and L2 larvae on squares (54.7%–60.2%) between DAS-21023-5 × DAS-24236-5 × SYN-IR102-7 and DAS-21023-5 × DAS-24236-5 (Table 2). Both GM cotton lines provided significantly greater mortality to *H*. *armigera* compared to the non-Bt cotton on all tissue types (Table 2). Mortality observed for the non-Bt cotton treatments averaged 2.3% or lower across tissues, indicating the bioassay test system was robust (Table 2).

**Table 2:** Percent mortality of neonates (on leaf disk) and L2 larvae (on square) of *H*. *armigera*, *H*. *zea* and the F_1_ progeny from ♀*H*. *armigera* × ♂*H*. *zea* after 5 d on leaves of Bt and non-Bt cotton technologies in laboratory trials.

Pest species and cotton technologies	*n* ^*a*^	Leaf disk (growth stage 15)^b^		Leaf disk (growth stage 55)^b^		Square (growth stage 55)^b^	
		Dead	% mortality (95% CI)	Dead	% mortality (95% CI)	Dead	% mortality (95% CI)
***Helicoverpa armigera* (Ha-Lab)**							
DAS-21023-5 × DAS-24236-5 × SYN-IR102-7	128	110	85.9 (79.0–91.1) a	92	71.9 (63.6–79.1) a	70	54.7 (46.0–63.1) a
DAS-21023-5 × DAS-24236-5	128	93	72.7 (64.4–79.8) a	86	67.2 (58.7–74.9) a	77	60.2 (51.5–68.3) a
Non-Bt cotton	128	3	2.3 (0.7–6.5) b	3	2.3 (0.7–6.5) b	1	0.8 (0.1–4.4) b
***Helicoverpa zea* (Hz-Lab)**							
DAS-21023-5 × DAS-24236-5 × SYN-IR102-7	128	128	100.0 (97.2–100.0) a	128	100.0 (97.2–100.0) a	128	100.0 (97.2–100.0) a
DAS-21023-5 × DAS-24236-5	128	128	100.0 (97.2–100.0) a	53	41.4 (33.1–50.0) b	70	54.7 (46.0–63.1) b
Non-Bt cotton	128	16	12.5 (7.6–19.2) b	10	7.8 (4.1–13.6) c	14	10.9 (6.4–17.3) c
**F**_**1**_ **hybryd (♀ Ha-Lab × ♂ Hz-Lab)**^**c**^							
DAS-21023-5 × DAS-24236-5 × SYN-IR102-7	128	128	100.0 (97.2–100.0) a	97	75.8 (67.8–82.3) a	48	37.5 (29.5–46.1) a
DAS-21023-5 × DAS-24236-5	128	59	46.1 (37.6–54.8) b	51	39.8 (31.7–48.5) b	15	11.7 (7.0–18.2) b
Non-Bt cotton	128	14	10.9 (6.4–17.3) c	16	12.5 (7.6–19.2) c	3	2.3 (0.7–6.5) c

^a^Number of larvae tested per growth stage.

^b^Growth stage number according to Munger et al. [28]. Means (95% CI) followed by the same letter in each column and species are not significantly different due to nonoverlap of 95% CIs.

^c^Only the crossing of ♀*H*. *armigera* × ♂*H*. *zea* produced fertile eggs allowing bioassays to be carried out.

No surviving *H*. *zea* neonates (Hz-Lab) were also detected at 5 d on leaf-disks harvested at growth stage 15 of DAS-21023-5 × DAS-24236-5 × SYN-IR102-7 and DAS-21023-5 × DAS-24236-5 cotton technologies (Table 3). In contrast, on leaf-disk of DAS-21023-5 × DAS-24236-5 from growth stage 55 (neonates) and on squares (L2 larvae) of this same growth stage, mortality (≤54.7%) was significantly lower than on DAS-21023-5 × DAS-24236-5 × SYN-IR102-7 (Table 2). On non-Bt cotton, the mortalities of neonates (leaf-disk) and L2 larvae (squares) of *H*. *zea* was ≤12.5%.

**Table 3:** Damage caused by neonates (on leaf disk) and L2 larvae (on square) of *H*. *armigera*, *H*. *zea* and the F_1_ progeny from ♀*H*. *armigera* × ♂*H*. *zea* after 5 d on leaves of Bt and non-Bt cotton technologies in laboratory trials.

Pest species and cotton technologies	Damage on leaf disks (growth stage 15)^a^		Damage on leaf disks (growth stage 55)^a^		Damage on square (growth stage 55)^a^		
	Consumed leaf area (cm^2^)	% leaf consumption	Consumed leaf area (cm^2^)	% leaf consumption	% squares with surface feeding only	% larvae bored into square	% squares with any damage
***Helicoverpa armigera* (Ha-Lab)**							
DAS-21023-5 × DAS-24236-5 × SYN-IR102-7	0.03 ± 0.01 b	1.9 ± 0.5 b	0.09 ± 0.01 b	4.6 ± 0.5 b	39.1 ± 6.4 ab	28.9 ± 3.5 b	67.9 ± 6.1 b
DAS-21023-5 × DAS-24236-5	0.07 ± 0.01 b	5.0 ± 0.5 b	0.09 ± 0.02 b	3.4 ± 0.5 b	11.7 ± 3.8 c	57.8 ± 7.1 a	70.3 ± 4.2 b
Non-Bt cotton	1.13 ± 0.01 a	60.3 ± 3.0 a	1.0 ± 0.03 a	52.2 ± 1.7 a	30.1 ± 2.2 b	68.8 ± 2.4 a	99.2 ± 0.8 a
***Helicoverpa zea* (Hz-Lab)**							
DAS-21023-5 × DAS-24236-5 × SYN-IR102-7	0.0 ± 0.0 b	0.0 ± 0.0 b	0.0 ± 0.0 c	0.0 ± 0.0 c	0.0 ± 0.0 c	0.0 ± 0.0 c	0.0 ± 0.0 c
DAS-21023-5 × DAS-24236-5	0.0 ± 0.0 b	0.0 ± 0.0 b	0.1 ± 0.01 b	6.4 ± 0.5 b	48.4 ± 6.1 a	26.9 ± 6.8 b	78.1 ± 3.7 b
Non-Bt cotton	0.4 ± 0.1 a	17.4 ± 1.0 a	0.3 ± 0.01 a	16.6 ± 0.7 a	28.9 ± 5.4 b	66.4 ± 5.9 a	94.5 ± 2.2 a
**F**_**1**_ **hybryd (♀ Ha-Lab × ♂ Hz-Lab)**							
DAS-21023-5 × DAS-24236-5 × SYN-IR102-7	0.0 ± 0.0 b	0.0 ± 0.0 c	0.4 ± 0.01 b	2.4 ± 0.8 c	49.2 ± 5.2 a	46.1 ± 4.7 c	94.5 ± 2.5 a
DAS-21023-5 × DAS-24236-5	0.12 ± 0.03 b	9.6 ± 1.6 b	0.11 ± 0.01 b	6.3 ± 0.7 b	32.8 ± 3.1 b	60.2 ± 4.2 b	93.0 ± 2.2 a
Non-Bt cotton	0.43 ± 0.03 a	23.2 ± 1.5 a	0.41 ± 0.04 a	21.8 ± 2.0 a	16.4 ± 3.7 c	82.8 ± 4.0 a	99.2 ± 0.8 a

^a^Growth stage number according to Munger et al. [28]. Means ± SE followed by the same letter in each column and species are not significantly different as determined by the Tukey’s test at α = 0.05.

For F_1_ hybrids from ♀ Ha-Lab × ♂ Hz-Lab, a significantly higher mortality was observed for neonates at 5 d post-exposure on leaf-disks (75.8%–100%) and L2 larvae on floral buds (37.5%) of DAS-21023-5 × DAS-24236-5 × SYN-IR102-7 compared to DAS-21023-5 × DAS-24236-5 (39.8%–46.1% on leaf-disks and 11.7% on squares) and non-Bt cotton (≤12.5%) (Table 2).

#### Damage on leaf-disk and squares

Surviving neonates of *H*. *armigera* (Ha-Lab) at 5 d on leaf-disks of both cotton growth stages evaluated of DAS-21023-5 × DAS-24236-5 × SYN-IR102-7 and DAS-21023-5 × DAS-24236-5 caused significantly lower damage (≤5% of leaf consumption) than on non-Bt cotton (>52% of leaf consumption) (growth stage 15: *F* = 359.59; *df*_(Num/Den)_ = 2/21; *P* < 0.0001 and growth stage 55: *F* = 666.26; *df*_(Num/Den)_ = 2/21; *P* < 0.0001) (Table 3). At the same time, on DAS-21023-5 × DAS-24236-5 × SYN-IR102-7, a lower number of L2 larvae of *H*. *armigera* bored into squares (28.9%) and squares were less damaged (67.9%) than on non-Bt cotton (68.8% of larvae bored into squares with 99.2% of squares presenting any damage) (for both growth stages and each variable *df*_(Num/Den)_ = 2/21; *P* < 0.0001) (Table 3). Still, the number of squares with surface feeding was significantly higher for DAS-21023-5 × DAS-24236-5 × SYN-IR102-7 than DAS-21023-5 × DAS-24236-5, indicating that a low number of surviving larvae bored into squares of DAS-21023-5 × DAS-24236-5 × SYN-IR102-7 cotton, and remained feeding on the surface (*F* = 16.44; *df*_(Num/Den)_ = 2/21; *P* = 0.0011) (Table 3).

The surviving *H*. *zea* neonates (Hz-Lab) on leaf-disks at growth stages 15 and 55 of DAS-21023-5 × DAS-24236-5 (only on this Bt cotton had survivors) had less than 7% of leaf consumption differing significantly from damage caused by larvae on non-Bt cotton (16.6% and 17.4% of leaf consumption) (growth stage 15: *F* = 357.29; *df*_(Num/Den)_ = 2/21; *P* < 0.0001 and growth stage 55: *F* = 250.06; *df*_(Num/Den)_ = 2/21; *P* < 0.0001) (Table 3). Similarly, on DAS-21023-5 × DAS-24236-5 cotton had a lower number of L2 larvae of *H*. *zea* bored into squares (26.9%) and less squares presented damage (78%) than on non-Bt cotton (66.4% of larvae bored into squares and 94.5% of squares presented damage) (for each variable *df*_(Num/Den)_ = 2/21; *P* < 0.0001) (Table 3). In addition, squares with only surface feeding were higher on DAS-21023-5 × DAS-24236-5 cotton (48.4%) than on non-Bt cotton (28.9%), indicating that surviving L2 *H*. *zea* larvae had pronounced growth inhibition on this Bt cotton technology, affecting its capacity to bore into squares (*F* = 408.31; *df*_(Num/Den)_ = 2/21; *P* < 0.0001) (Table 3).

When F_1_ hybrids from ♀ Ha-Lab × ♂ Hz-Lab were evaluated, significantly lower damage from surviving larvae on leaf-disks of both growth stages of DAS-21023-5 × DAS-24236-5 × SYN-IR102-7 and DAS-21023-5 × DAS-24236-5 cotton events (≤9.6% leaf consumption) than on non-Bt cotton (21.8%–23.3% of leaf consumption) were detected (growth stage 15: *F* = 87.25; *df*_(Num/Den)_ = 2/21; *P* < 0.0001 and growth stage 55: *F* = 59.48; *df*_(Num/Den)_ = 2/21; *P* < 0.0001) (Table 3). Similarly, DAS-21023-5 × DAS-24236-5 × SYN-IR102-7 had significantly higher squares with surface feeding (49.2%) and a lower number of larvae bored into squares (46.1%) compared to DAS-21023-5 × DAS-24236-5 (32.8% and 60.2%, respectively) and non-Bt cotton (16.4% and 82.8%, respectively), demonstrating that in three-gene Bt cotton, fewer larvae were able to bore into squares (for each variable *df*_(Num/Den)_ = 2/21; *P* < 0.0001) (Table 3). Regardless, no significant differences in the percentage of squares with any damage among Bt and non-Bt cotton treatments evaluated (93%–99.2%) were detected (*F* = 2.74; *df*_(Num/Den)_ = 2/21; *P* = 0.0879) (Table 3).

Surviving neonates (on leaf-disk) and L2 larvae (on squares) of *H*. *armigera* (Ha-Lab) and F_1_ hybrids on DAS-21023-5 × DAS-24236-5 × SYN-IR102-7 and DAS-21023-5 × DAS-24236-5 cotton technologies had significant lower larval weights (<0.90 mg/larva on leaf-disk and <3.7mg/larva on floral buds) than on non-Bt cotton (>4.6 mg/larva on leaf-disk and >12.6 mg/larva on floral buds) (for each pest, growth stage and plant structure *df*_(Num/Den)_ = 2/21; *P* < 0.0001). These results indicate that surviving larvae on Bt cotton technologies had more than 80% (on leaf-disk) and 70% (on squares) growth inhibition relative to those fed on non-Bt cotton. For *H*. *zea* (Hz-Lab), the larval weights were not computed due to the limited number of insects surviving on both Bt cotton technologies.

### B. Survivorship of *Helicoverpa* species on Bt cotton throughout the larval cycle

The survivorship of *H*. *armigera* (Ha-Lab) decreased with increased feeding time on leaves of DAS-21023-5 × DAS-24236-5 × SYN-IR102-7 and DAS-21023-5 × DAS-24236-5 cotton technologies (Table 4). For *H*. *armigera*, significant lower survivorship from 5 d onwards on DAS-21023-5 × DAS-24236-5 × SYN-IR102-7 and DAS-21023-5 × DAS-24236-5 DAS-21023-5 × DAS-24236-5 than on non-Bt cotton, with <1% of larvae surviving until 30 d on leaves of both Bt cotton technologies. In the same period, on non-Bt cotton 52.2% of larvae developed into L5 and L6 stages (Table 4).

**Table 4:** Survivorship of *H*. *armigera* (Ha-Lab) and *H*. *zea* (Hz-Lab) on leaves of Bt and non-Bt cotton technologies throughout the larval cycle in laboratory trials.

Feeding time (d)	DAS-21023-5 × DAS-24236-5 × SYN-IR102-7^a^		DAS-21023-5 × DAS-24236-5^a^		Non-Bt cotton^a^	
	*n*	% survival (95% CI)	*n*	% survival (95% CI)	*n*	% survival (95% CI)
***Helicoverpa armigera* (Ha-Lab)**						
0	420	100.0 (99.2–100.0) a	260	100.0 (98.6–100.0) a	130	100.0 (97.3–100.0) a
5	104	25.0 (21.0–29.3) b	112	42.4 (36.6–48.4) b	121	89.0 (82.8–93.4) b
10	46	11.1 (8.3–14.4) c	76	28.8 (23.6–34.5) c	112	82.3 (75.2–88.0) b
15	23	5.5 (3.7–8.1) d	25	9.5 (6.4–13.5) d	91	67.0 (58.7–74.4) c
20	13	3.1 (1.8–5.2) de	10	3.8 (2.0–6.7) de	84	61.8 (53.4–69.6) c
25	8	2.0 (0.9–3.7) de	3	1.1 (0.3–3.2) ef	75	55.1 (46.7–63.3) c
30	3	0.7 (0.2–2.1) e	0	0.0 (0.0–1.4) f	71	52.2 (43.8–60.5) c
***Helicoverpa zea* (Hz-Lab)** ^b^						
0	420	100.0 (99.2–100.0) a	260	100.0 (98.6–100.0) a	130	100.0 (97.3–100.0) a
5	0	0.0 (0.0–0.01) b	151	58.1 (52.0–63.9) b	111	85.4 (78.5–90.6) b

^a^Means (95% CI) followed by the same letter in each column for each species and cotton variety are not significantly different due to nonoverlap of 95% CIs.

^b^The evaluations were performed only up to 5 d, as there was complete mortality of the Hz-Lab population on leaves of DAS-21023-5 × DAS-24236-5 × SYN-IR102-7 cotton.

For larvae of *H*. *zea* (Hz-Lab), no survivors after 5 d on leaves of DAS-21023-5 × DAS-24236-5 × SYN-IR102-7 were observed (Table 4). Nonetheless, surviving larvae on DAS-21023-5 × DAS-24236-5 presented 81% stunting (larvae that did not reach L2 stage) when compared to those developed on non-Bt cotton. At the same time point, 58.1 and 85.4% survival were verified on DAS-21023-5 × DAS-24236-5 and non-Bt cotton, respectively.

In contrast, the population of *H*. *zea* (Hz-field) collected during 2021/22 season surviving on Bt cotton expressing Cry1Ab, Cry2Ae and Vip3Aa proteins, at F_1_ generation, showed 37% survival on leaves of DAS-21023-5 × DAS-24236-5 × SYN-IR102-7 and 68% on DAS-21023-5 × DAS-24236-5 at 30 d, developing into L5 and L6 stages (Table 5). On non-Bt cotton, the survival rate was 84% at the same period.

**Table 5:** Survivorship of *H*. *zea* from Bahia (Hz-field) on leaves of Bt and non-Bt cotton technologies throughout the larval cycle in laboratory trials.

Feeding time (d)	DAS-21023-5 × DAS-24236-5 × SYN-IR102-7^a^		DAS-21023-5 × DAS-24236-5^a^		Non-Bt cotton^a^	
	*n*	% survival (95% CI)	*n*	% survival (95% CI)	*n*	% survival (95% CI)
***Helicoverpa zea* (Hz-field from Bahia)**						
0	100	100.0 (96.4–100.0) a	100	100.0 (96.4–100.0) a	100	100.0 (96.4–100.0) a
5	100	100.0 (96.4–100.0) a	100	100.0 (96.4–100.0) a	100	100.0 (96.4–100.0) a
10	89	89.0 (81.6–94.0) a	100	100.0 (96.4–100.0) a	100	100.0 (96.4–100.0) a
15	79	79.0 (70.2–86.1) a	100	100.0 (96.4–100.0) a	100	100.0 (96.4–100.0) a
20	73	73.0 (63.7–81.0) b	98	98.0 (93.1–99.6) a	100	100.0 (96.4–100.0) a
25	62	62.0 (52.2–71.1) b	84	84.0 (75.8–90.1) b	96	96.0 (90.4–98.6) ab
30	37	37.0 (28.0–46.8) c	68	68.0 (58.4–76.5) b	84	84.0 (75.8–90.1) b

^a^Means (95% CI) followed by the same letter in each column for each species and cotton variety are not significantly different due to nonoverlap of 95% CIs.

### C. Field trials

**Fig 1:**
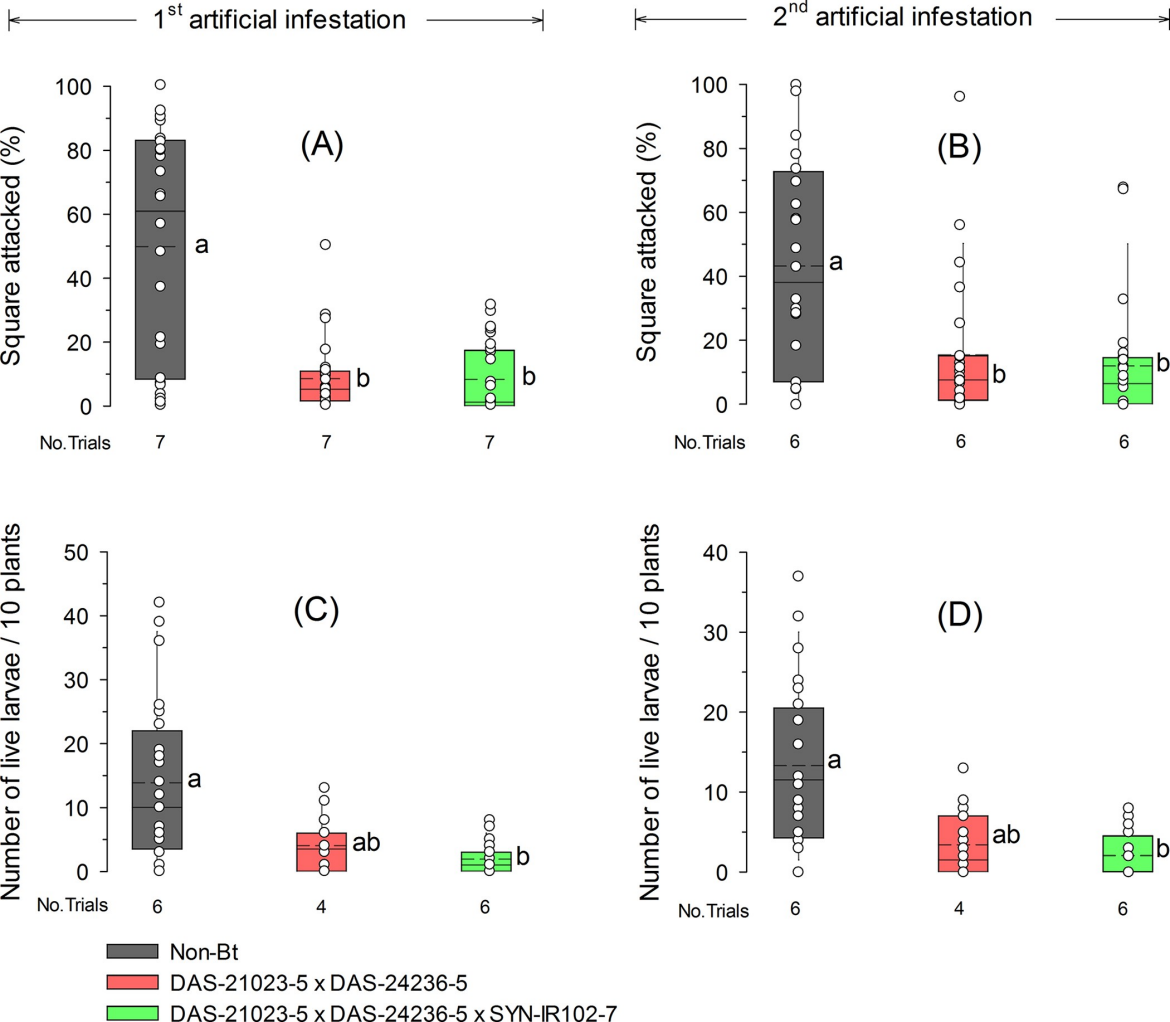
Squares attacked (A—B) and live larvae (C—D) of *Helicoverpa armigera* in Bt and non-Bt cotton technologies after two field artificial infestations (L1 stage) during the cotton reproductive growth stage. The dashed and solid lines in boxplots show the mean and median across trials, respectively. Dot markers indicate values from individual field trial plots. Boxplots with different letters were significant different by a Tukey’s test (α = 0.05).

The Bt cotton technologies with the events DAS-21023-5 × DAS-24236-5 ×SYN-IR102-7 and DAS-21023-5 × DAS-24236-5 provided similar efficacy against *H*. *armigera* after artificial infestations under field conditions (Fig 1). Both Bt technologies significantly reduced the damaged squares compared to non-Bt cotton (1^st^ infestation: *F* = 13.3; *df*_(Num/Den)_ = 2/12; *P* < 0.001; 2^nd^ infestation: *F* = 10.5; *df*_(Num/Den)_ = 2/10; *P* = 0.004). In non-Bt cotton, the mean percentage of squares attacked by *H*. *armigera* was 49.9% after first infestation and 43.2% after second infestation, while in Bt cotton, squares attacked reached 8.6% and 15.5% for DAS-21023-5 × DAS-24236-5 and 8.4% and 11.9% for DAS-21023-5 × DAS-24236-5 × SYN-IR102-7 in the first and second infestation (Fig 1A and 1B). The mean number of *H*. *armigera* live larvae found on DAS-21023-5 × DAS-24236-5 × SYN-IR102-7 after the first (1.9 larvae/10 plants) and second infestation (2.0 larvae/10 plants) were significantly lower than on non-Bt cotton (13.9 and 13.3 larvae/10 plants, respectively) (1^st^ infestation: *F* = 7.5; *df*_(Num/Den)_ = 2/14.5; *P* = 0.005; 2^nd^ infestation: *F* = 8.4; *df*_(Num/Den)_ = 2/9.6; *P* = 0.007). However, both treatments did not differ from DAS-21023-5 × DAS-24236-5 (4.1 and 3.4 larvae/10 plants) in the two infestations (Fig 1C and 1D).

**Fig 2:**
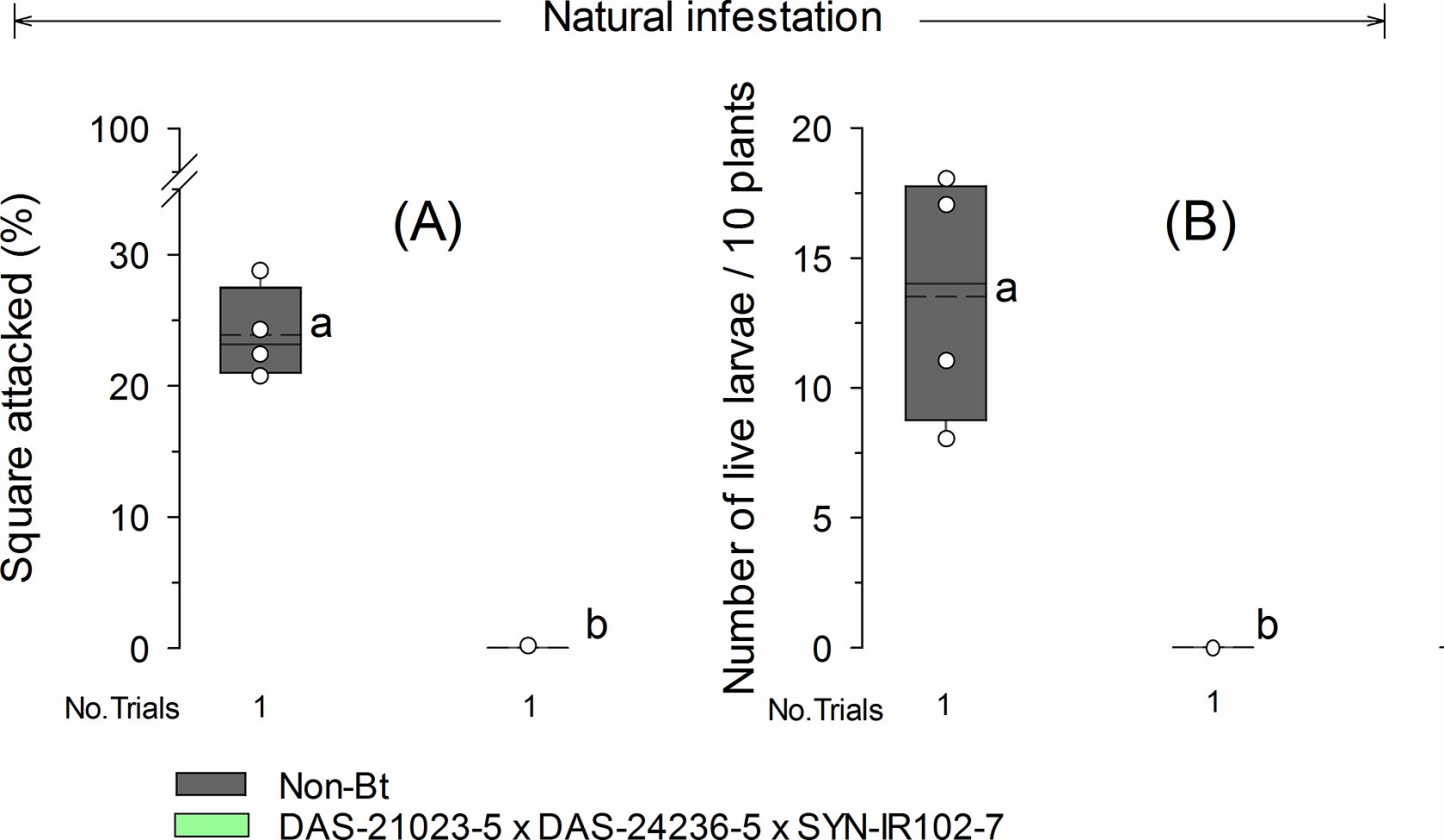
Squares attacked (A) and live larvae (B) of *Helicoverpa* spp. in Bt cotton technology DAS-21023-5 × DAS-24236-5 × SYN-IR102-7 and non-Bt cotton under field natural infestation during the cotton reproductive growth stage. The dashed and solid lines in boxplots show the mean and median, respectively. Dot markers indicate values from individual field trial plots. Boxplots with different letters were significant different by a paired *t*-test (α = 0.05).

Under a natural infestation of *Helicoverpa* spp., Bt cotton DAS-21023-5 × DAS-24236-5 × SYN-IR102-7 had significantly lower damage to squares (*t* = 47.2; *df* = 3; *P* < 0.001) and a lower number of live larvae (*t* = 8.5; *df* = 3; *P* = 0.003) compared to the non-Bt cotton (Fig 2). No square damage and live larvae of *Helicoverpa* spp. were observed on DAS-21023-5 × DAS-24236-5 × SYN-IR102-7, while in non-Bt cotton the mean percentage of square attack by natural infestation of *Helicoverpa* spp. was 23.8%, with 13.5 live larvae/10 plants (Fig 2A and 2B).

## Discussion

The susceptibility of *Helicoverpa* species to Cry1F and Vip3Aa19 in Brazil have not been fully characterized; susceptibility to another Vip protein (e.g. Vip3Aa20) was reported for *H*. *armigera* and *H*. *zea* and to Cry1Ac for *H*. *armigera*, indicating that *H*. *armigera* was more tolerant to Vip3Aa20 than *H*. *zea*, but had high susceptibility to Cy1Ac [35, 36]. To assess the performance of Cry1Ac, Cry1F and Vip3Aa19 Bt proteins expressed in DAS-21023-5 × DAS-24236-5 × SYN-IR102-7 cotton technology against the Heliothine species complex in Brazil, we performed laboratory and field studies with *H*. *armigera* and *H*. *zea* and the hybrid progeny of the species. In most experiments presented here, insects from laboratory colonies that were annually augmented with field-collected insects were used.

The relative high mortality levels observed for *Helicoverpa* species from laboratory-reared colonies and hybrids on leaves of DAS-21023-5 × DAS-24236-5 × SYN-IR102-7 cotton indicate that Cry1Ac, Cry1F and Vip3Aa19 are effective in protecting cotton plants against damage from these target pests. This 3-gene Bt cotton technology also improved protection against square damage caused by *Helicoverpa* species and hybrids compared to 2-gene Bt cotton and should improve yield protection, since larvae may prefer to attack reproductive structures of cotton [37, 38]. According to Rios et al. [7], there is chance of interspecific crosses in *Helicoverpa* species, with interspecific crossing probably occurring when pest populations are at high densities, suggesting that populations of these pests should be maintained low levels in the field. Therefore, the effectiveness of DAS-21023-5 × DAS-24236-5 × SYN-IR102-7 cotton in controlling *Helicoverpa* species may reduce population levels across the landscape, favoring that the ‘species identities’ will be maintained under field conditions.

When comparing survival of *H*. *zea* colonies throughout the larval life cycle in two different studies (Tables 4 and 5), the field population of *H*. *zea* collected in Bt cotton expressing Cry1Ab, Cry2Ae and Vip3A in western Bahia, Brazil during the 2021/2022 season had lower mortality on DAS-21023-5 × DAS-24236-5 × SYN-IR102-7 cotton technology than was observed for the laboratory *H*. *zea* population. During this season, it was verified a high occurrence of larvae of the genus *Helicoverpa* spp. in the western Bahia, Brazil causing considerable damage in reproductive structures of Bt cotton expressing Cry1Ab, Cry2Ae and Vip3Aa [39]. These larvae were later identified by PCR tests as *H*. *zea* and was the source of Hz-field population here tested. Our findings suggest that the Hz-field population collected from a Bt cotton field expressing Cry1, Cry2 and Vip3 in Bahia has lower susceptibility to Cry1 and Vip3Aa in comparison to the Hz-Lab. While not a definitive test for resistance to the Bt proteins (a known laboratory-susceptible *H*. zea population was not tested in the same study as the Bahia-collected Hz-field population), the current evidence (collection of survivors in a Bt field and the laboratory bioassay findings relative to other findings with a *H*. zea laboratory population) implies that reduced control is possible in the field. Diminished susceptibility to Bt proteins in *H*. *zea* and *H*. *armigera* has been previously reported in several states of U.S., India, Pakistan, and China [8–13, 40–42]. These observations reinforce the need for compliance with non-Bt refuge guidelines when planting Bt cotton technologies to retard the evolution of resistance in *Helicoverpa* species in Brazil.

Further investigation of *Helicoverpa* populations from the Bahia cotton-producing region are needed to fully understand the long-term implications for the durability of both Bt cotton and Bt maize against these species. For example, it will be important to confirm whether the Hz-field population is resistant to Cry1, Cry2 and/or Vip3Aa Bt proteins and to understand the performance of Hz-field on Bt maize hybrids. The ability of *H*. *zea* and *H*. *armigera* to produce hybrid offspring may also have implications for the development of resistance to Bt proteins in cotton-infesting populations of *H*. *armigera* that are not currently understood.

Currently, relevant exposure to Cry and Vip3Aa proteins by *Helicoverpa* species, which is part of DAS-21023-5 × DAS-24236-5 × SYN-IR102-7, is ongoing in Brazil, because of the widespread planting of Bt soybean, Bt cotton and Bt maize expressing a single-mode-of-action Bt proteins. Although *H*. *armigera* was first identified in soybean in Brazil [1], this species prefers cotton as a host plant [4]. Despite the relative high level of adoption of Bt cotton technologies expressing Cry proteins (~90% of the total cotton area), these technologies continue to provide reasonable levels of control against *Helicoverpa* species. The abundance of *Helicoverpa* species has not been quantified in cotton in Brazil, but studies stated a low abundance of *H*. *armigera* in soybean fields [20]. The low abundance of this pest in soybean is a positive aspect for the IPM, since same Bt proteins are expressed in cotton and soybean against *H*. *armigera*, indicating that broad adoption of Bt plants reduced population levels of this species.

From pest management perspective, the cotton technology DAS-21023-5 × DAS-24236-5 × SYN-IR102-7 beyond reasonable levels of mortality against *H*. *armigera*, also reduced the damage to squares. These results expand those previously reported by Marques et al. [25], showing this same Bt cotton technology is highly effective against other lepidopteran pests of cotton. It is noteworthy that, the non-Vip3Aa containing products usually did not provide full control of *S*. *frugiperda*, and growers implemented multiple insecticide sprays that probably contributed to controlling *Helicoverpa* spp. as well. As growers are moving to Vip3Aa-containing varieties, less insecticide sprays are needed for lepidopteran control, reducing the monitoring of lepidopterans and increasing potential for escapes of *Helicoverpa* spp. in the field [39]. Therefore, to maintain the benefits of DAS-21023-5 × DAS-24236-5 × SYN-IR102-7 cotton for pest control in Brazil, where *Helicoverpa* species have numerous generations per year and are exposed to same mode-of-action Bt proteins in other crops, this GM cotton technology should be integrated with other control tactics, including the adoption of at least 20% of structured refuge (non-Bt cotton) for resistance management.

## References

[1] Czepak C, Albernaz KC, Vivan LM, Guimarães HO, Carvalhais T. First reported occurrence of *Helicoverpa armigera* (Hübner) (Lepidoptera: Noctuidae) in Brazil. Pesqui Agropecu Trop. 2013; 43: 110–113.

[2] Pinto FA, Mattos MVV, Silva FWS, Rocha SL, Elliot SL. The spread of *Helicoverpa armigera* (Lepidoptera: Noctuidae) and coexistence with *Helicoverpa zea* in Southeastern Brazil. Insects. 2017; 8: 87. 28869528 10.3390/insects8030087 PMC5620707

[3] Gonçalves RM, Mastrangelo TA, Rodrigues JCV, Paulo DF, Omoto C, Corrêa AS, et al. Invasion origin, rapid population expansion, and the lack of genetic structure of cotton bollworm (*Helicoverpa armigera*) in the Americas. Ecol Evol. 2019; 9: 7378–7401. 31346410 10.1002/ece3.5123 PMC6635935

[4] Dourado PM, Pantoja-Gomez LM, Horikoshi RJ, Carvalho RA, Omoto C, Corrêa AS, et al. Host plant use of *Helicoverpa* spp. (Lepidoptera: Noctuidae) in the Brazilian agricultural landscape. Pest Manag Sci. 2021; 77: 780–794. 32902104 10.1002/ps.6079

[5] Anderson CJ, Oakeshott JG, Tay WT, Gordon KHJ, Zwick A, Walsh TK. Hybridization and gene flow in the mega-pest lineage of moth, *Helicoverpa*. Proc Natl Acad Sci USA. 2018; 115: 5034–5039. 29610329 10.1073/pnas.1718831115 PMC5948968

[6] Cordeiro EMG, Pantoja-Gomez LM, Paiva JB, Nascimento ARB, Omoto C, Michel AP, et al. Hybridization and introgression between *Helicoverpa armigera* and *H*. *zea*: an adaptational bridge. BMC Evol Biol. 2020; 20: 61. 32450817 10.1186/s12862-020-01621-8 PMC7249340

[7] Rios DAM, Specht A, Roque-Specht VF, Sosa-Gómez DR, Fochezato J, Malaquias JV, et al. *Helicoverpa armigera* and *Helicoverpa zea* hybridization: constraints, heterosis, and implications for pest management. Pest Manag Sci. 2021; 78: 955–964. 34729903 10.1002/ps.6705

[8] Tabashnik BE. ABCs of insect resistance to Bt. PLoS Genet. 2015; 11: e1005646. doi: 10.1371/journal.pgen.1005646 26583776 PMC4652897

[9] Dively GP, Venugopal PD, Finkenbinder C. Field-evolved resistance in corn earworm to Cry proteins expressed by transgenic sweet corn. PLoS One. 2016; 11: e0169115. doi: 10.1371/journal.pone.0169115 28036388 PMC5201267

[10] Reisig DD, Huseth AS, Bacheler JS, Aghaee MA, Braswell L, Burrack HJ, et al. Long-term empirical and observational evidence of practical *Helicoverpa zea* resistance to cotton with pyramided Bt toxins. J Econ Entomol. 2018; 111: 1824–1833. 29668958 10.1093/jee/toy106

[11] Yang F, Kerns DL, Little N, Brown SA, Stewart SD, Catchotd AL, et al. Practical resistance to Cry toxins and efficacy of Vip3Aa in Bt cotton against *Helicoverpa zea*. Pest Manag Sci. 2022; 78: 5234–5242. 36053801 10.1002/ps.7142

[12] Dandan Z, Yutao X, Wenbo C, Yanhui L, Kongming W. Field monitoring of *Helicoverpa armigera* (Lepidoptera: Noctuidae) Cry1Ac insecticidal protein resistance in China (2005–2017). Pest Manag Sci. 2019; 75: 753–759. 30101444 10.1002/ps.5175

[13] Singh TVK, Kukanur VS, Supriya GB. Frequency of resistance alleles to Cry1Ac toxin from cotton bollworm, *Helicoverpa armigera* (Hübner) collected from Bt-cotton growing areas of Telangana state of India. J Invertebr Pathol. 2021; 183: 107559. 33617874 10.1016/j.jip.2021.107559

[14] Leite NA, Alves-Pereira A, Corrêa AS, Zucchi MI, Omoto C. Demographics and genetic variability of the New World bollworm (*Helicoverpa zea*) and the Old World bollworm (*Helicoverpa armigera*) in Brazil. PLoS One. 2014; 9: e113286. 25409452 10.1371/journal.pone.0113286 PMC4237417

[15] Bentivenha JPF, Paula-Moraes SV, Baldin ELL, Specht A, Silva IF, Hunt TE. Battle in the New World: *Helicoverpa armigera* versus *Helicoverpa zea* (Lepidoptera: Noctuidae). PLoS One. 2016; 11: e0167182. 27907051 10.1371/journal.pone.0167182 PMC5132268

[16] Blanco CA, Chiaravalle C, Dalla-Rizza M, Farias JR, García-Degano MF, Gataminza G, et al. Current situation of pests targeted by Bt crops in Latin America. Curr Opin Insect Sci. 2016; 15: 131–138. doi: 10.1016/j.cois.2016.04.012 27436743

[17] Silva FR, Trujillo D, Bernardi O, Rodrigues JCV, Bailey WD, Gilligan TM. et al. Comparative toxicity of *Helicoverpa armigera* and *Helicoverpa zea* (Lepidoptera: Noctuidae) to selected insecticides. Insects. 2020; 11: 431. 32664300 10.3390/insects11070431 PMC7412147

[18] Lu Y, Wu K, Jiang Y, Guo Y, Desneux N. Widespread adoption of Bt cotton and insecticide decrease promotes biocontrol services. Nature. 2012; 487: 362–365. doi: 10.1038/nature11153 22722864

[19] Brookes G, Barfoot P. Farm income and production impacts of using GM crop technology 1996–2015. GM Crops & Food. 2018; 8: 156–193. 10.1080/21645698.2017.1317919 PMC5617554 28481684

[20] Horikoshi RJ, Dourado PM, Berger GU, Fernandes DS, Omoto C, Wilse A, et al. Large-scale assessment of lepidopteran soybean pests and efficacy of Cry1Ac soybean in Brazil. Sci Rep. 2021; 11: 15956. doi: 10.1038/s41598-021-95483-9 34354186 PMC8342623

[21] Céleres. 2019. Informativo de Biotecnologia Céleres. IB 19.01. Novembro de 2019. Available from: http://www.celeres.com.br/wp-content/uploads/2019/11/BoletimBiotecnologiaC%C3%A9leres_Novembro2019-2.pdf

[22] Horikoshi RJ, Bernardi D, Bernardi O, Malaquias JB, Okuma DM, Miraldo LL. et al. Effective dominance of resistance of *Spodoptera frugiperda* to Bt maize and cotton varieties: implications for resistance management. Sci Rep. 2016; 6: 34864. 27721425 10.1038/srep34864 PMC5056508

[23] Brévault T, Heuberger S, Zhang M, Ellers-Kirk C, Ni X, Masson L, et al. Potential shortfall of pyramided transgenic cotton for insect resistance management. Proc Natl Acad Sci USA. 2013; 110: 5806–5811. doi: 10.1073/pnas.1216719110 23530245 PMC3625267

[24] Carrière Y, Crickmore N, Tabashnik BE. Optimizing pyramided transgenic Bt crops for sustainable pest management. Nat Biotechnol. 2015; 33: 161–168. doi: 10.1038/nbt.3099 25599179

[25] Marques LH, Lepping M, Castro BA, Santos AC, Rossetto J, Nunes MZ, et al. Field efficacy of Bt cotton containing events DAS-21023-5 × DAS-24236-5 × SYN-IR102-7 against lepidopteran pests and impact on the non-target arthropod community in Brazil. PLoS One. 2021; 16: e0251134. 33945577 10.1371/journal.pone.0251134 PMC8096009

[26] Greene GL, Leppla NC, Dickerson WA. Velvetbean caterpillar: a rearing procedure and artificial medium. J Econ Entomol. 1976; 69: 487–488.

[27] Perera OP, Allen KC, Jain D, Purcell M, Little NS, Luttrell RG. Rapid identification of *Helicoverpa armigera* and *Helicoverpa zea* (Lepidoptera: Noctuidae) using ribosomal RNA internal transcribed spacer 1. J Insect Sci. 2015; 15: 155. 26516166 10.1093/jisesa/iev137 PMC4625950

[28] Munger P, Bleiholder H, Hack H, Hess M, Stauß R, Van den Boom T. et al. Phenological growth stages of the cotton plant (*Gossypium hirsutum L*.) codification and description according to the BBCH scale. J Agron Crop Sci. 2018; 180: 143–149.

[29] Dorai-Raj S. binom: Binomial confidence intervals for several parameterizations. R package version. 2009; 1.0–5. Available from: http://CRAN.R-project.org/package_binom

[30] R Development Core Team. R: a language and environment for statistical computing. R Foundation for Statistical Computing, Vienna, Austria; 2018.

[31] Savin NE, Robertson JL, Russell RM. A critical evaluation of bioassay in insecticide research: likelihood ratio tests of dose-mortality regression. Bull Entomol Soc Am. 1977; 23: 257–266.

[32] Robertson JL, Russel RM, Preisler HK, Savin NE. Bioassays with arthropods, 2nd ed. CRC Press, Boca Raton, FL; 2007.

[33] SAS Institute. Statistical analysis system: getting started with the SAS learning. SAS Institute, Cary, NC; 2008.

[34] Pimentel-Gomes F. Curso de estatística experimental. FEALQ, Piracicaba; 2009.

[35] Dourado PM, Bacalhau FB, Amado D, Carvalho RA, Martinelli S, Head GP, et al. High susceptibility to Cry1Ac and low resistance allele frequency reduce the risk of resistance of *Helicoverpa armigera* to Bt soybean in Brazil. PLoS One 2016; 11: e0161388. 27532632 10.1371/journal.pone.0161388 PMC4988708

[36] Leite NA, Pereira RM, Durigan MR, Amado D, Fatoretto J, Medeiros FCL, et al. Susceptibility of Brazilian populations of *Helicoverpa armigera* and *Helicoverpa zea* (Lepidoptera: Noctuidae) to Vip3Aa20. J Econ Entomol. 2018; 111: 399–404. 29272429 10.1093/jee/tox336

[37] Kumar S, Saini RK. Feeding preference and damage potential of *Helicoverpa armigera* (Hübner) on different promising cotton genotypes/hybrid. J Agr Sci Tech. 2008; 10: 411–420.

[38] Sivasupramaniam S, Moar WJ, Ruschke LG, Osborn JA, Jiang C, Sebaugh JL, et al. 2008. Toxicity and characterization of cotton expressing *Bacillus thuringiensis* Cry1Ac and Cry2Ab2 proteins for control of lepidopteran pests. J Econ Entomol. 2008; 101: 546–554. 18459423 10.1603/0022-0493(2008)101[546:tacoce]2.0.co;2

[39] Instituto Mato-Grossense do Algodão (IMAmt). *Helicoverpa* spp. alerta de monitoramento em lavouras de algodão Vip. 2022; Circular Técnica n.° 52. Available from: https://imamt.org.br/wp-content/uploads/2022/07/circular_tecnica_edicao52.pdf

[40] Ahmad S, Cheema HMN, Khan AA, Khan RSA, Ahmad JN. Resistance status of *Helicoverpa armigera* against Bt cotton in Pakistan. Transgenic Res. 2019; 28: 199–212. 30790127 10.1007/s11248-019-00114-9

[41] Yang F, Gonzáles JCS, Sword GA, Kerns DL. Genetic basis of resistance to the Vip3Aa Bt protein in *Helicoverpa zea*. Pest Manag Sci. 2020; 77: 1530–1535. 33201547 10.1002/ps.6176

[42] Santiago-González JC, Kerns DL, Yang F. Resistance allele frequency of *Helicoverpa zea to* Vip3Aa *Bacillus thuringiensis* protein in the Southeastern U.S. Insects. 2023; 14: 161. 36835730 10.3390/insects14020161 PMC9958976

